# Using a color-coded ambigraphic nucleic acid notation to visualize conserved palindromic motifs within and across genomes

**DOI:** 10.1186/1471-2164-15-52

**Published:** 2014-01-22

**Authors:** David A Rozak, Anthony J Rozak

**Affiliations:** 1 An independent investigator, Frederick, MD, USA; 2 Department of Visual Studies, College of Arts and Sciences, State University of New York at Buffalo, Buffalo, NY, USA

**Keywords:** Notation, Ambigram, Palindrome, Motif, Nucleotide, Color-coded

## Abstract

**Background:**

Ambiscript is a graphically-designed nucleic acid notation that uses symbol symmetries to support sequence complementation, highlight biologically-relevant palindromes, and facilitate the analysis of consensus sequences. Although the original Ambiscript notation was designed to easily represent consensus sequences for multiple sequence alignments, the notation’s black-on-white ambiguity characters are unable to reflect the statistical distribution of nucleotides found at each position. We now propose a color-augmented ambigraphic notation to encode the frequency of positional polymorphisms in these consensus sequences.

**Results:**

We have implemented this color-coding approach by creating an Adobe Flash® application ( http://www.ambiscript.org) that shades and colors modified Ambiscript characters according to the prevalence of the encoded nucleotide at each position in the alignment. The resulting graphic helps viewers perceive biologically-relevant patterns in multiple sequence alignments by uniquely combining color, shading, and character symmetries to highlight palindromes and inverted repeats in conserved DNA motifs.

**Conclusion:**

Juxtaposing an intuitive color scheme over the deliberate character symmetries of an ambigraphic nucleic acid notation yields a highly-functional nucleic acid notation that maximizes information content and successfully embodies key principles of graphic excellence put forth by the statistician and graphic design theorist, Edward Tufte.

## Background

**Figure 1:**
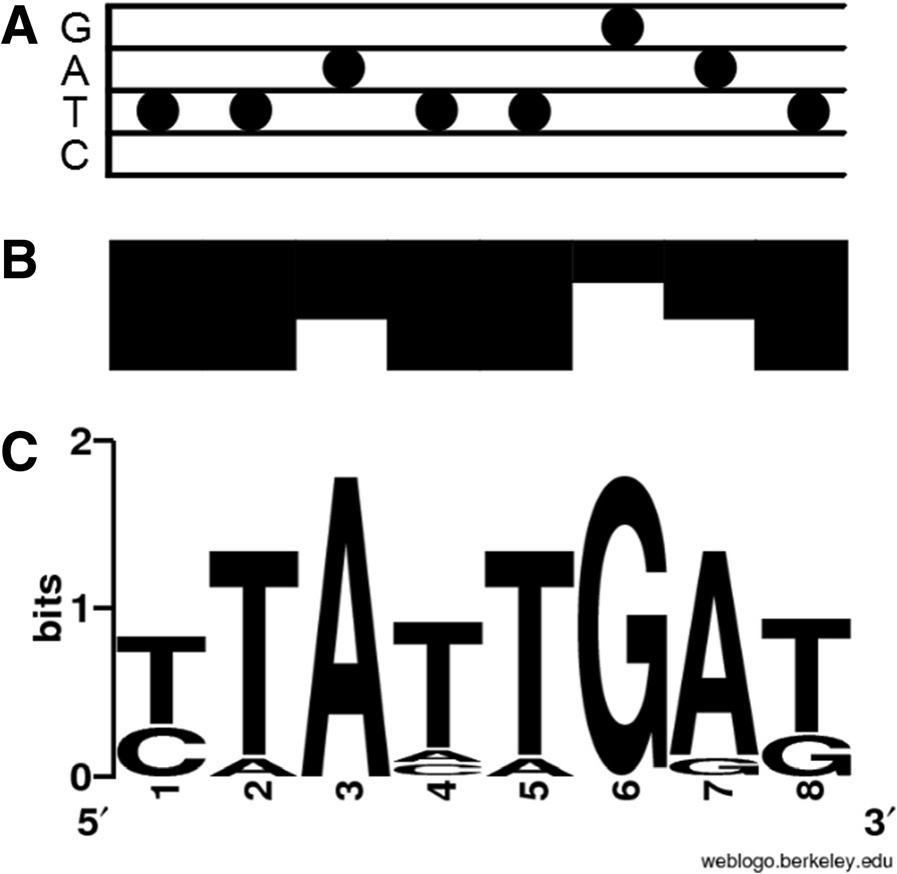
**Various strategies have been developed for visualizing genetic sequences.** For example, the notation proposed by Cowin et al. [3] uses dots arranged like notes on a music stave to display genetic data **(A)**. Jarvius and Landegren’s DNA Skyline notation [4] represents nucleotides as differently sized blocks **(B)**. Finally, Schneider and Stephens’ Sequence Logo [6] uses stacked, variably-sized IUPAC characters to display genetic variation in sequence alignments **(C)**.

The broadly applied International Union of Pure and Applied Chemistry’s (IUPAC) notation for nucleic acids was designed primarily as a short-hand for describing strings of bases [1,2]. Although representing each base with the first letter of its chemical name provides a convenient mnemonic for generating and interpreting genetic sequences, the Roman characters do little to support the visualization or manipulation of sequence data. Consequently, biologists have explored various graphic devices to augment the display and analysis of genetic data. For example, one such schema represents the bases as dots on a musical stave [3] (Figure 1A) and a second, more recent strategy displays nucleotides as a string of variably sized blocks [4] (Figure 1B). Both techniques offer the advantage of highlighting sequence polymorphisms but do not support clear visualization of consensus sequences from multiple sequence alignments.

**Figure 2:**
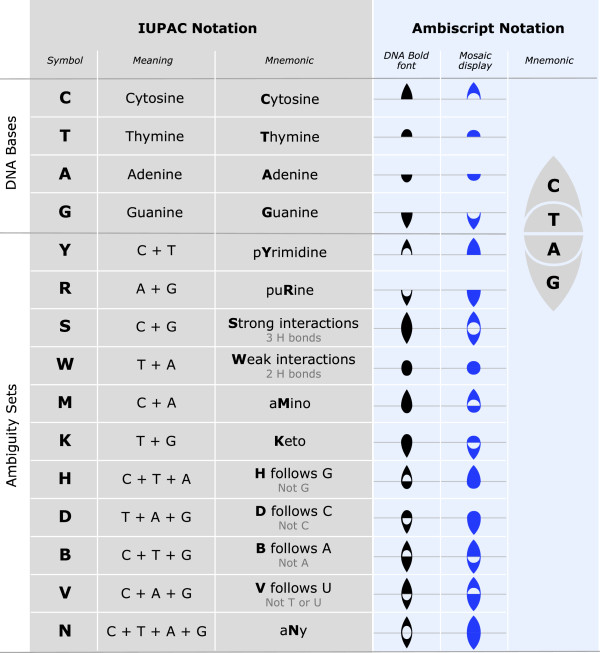
**Ambiscript Mosaic offers improvements over the traditional IUPAC and original Ambiscript nucleic acid notation.** As with the orignal Ambiscrpt DNA Bold font, the Ambiscript Mosaic symbols facilitate reverse complementation and the derivation of ambiguity characters via compounded symbols. However, by using nested symbols rather than figure-ground-reversals to construct the eleven ambiguity characters, Ambiscript Mosaic also supports the creation of color-coded consensus sequences. Furthermore, when read from top to bottom, the nested Ambiscript Mosaic symbols correspond to the IUPAC characters C, T, A, and G (or "sea tag"), providing an easy mnemonic for the meaning of the ambigraphic symbols.

The IUPAC first tackled the issue of consensus sequences in 1984 when they expanded the existing alphabetic notation system to encode positional variation [2]. The IUPAC ambiguity code recruited eleven additional letters from the Roman alphabet to indicate positions in a consensus sequence where two, three, or four bases were found among alignments of structurally and functionally homologous sequences (Figure 2). In 2010, Johnson proposed further expanding this code by enlisting additional Roman characters to encode nucleotide preference in a consensus sequence [5]. Despite their practical dependence on widely available ASCII characters, the main drawback with both approaches for creating consensus sequences is that the expanded code is complex and often difficult to interpret.

The sequence logo, developed by Schneider and Stephens in 1990 [6] (Figure 1C) is another frequently-used strategy for visualizing multiple sequence alignments. Schneider and Stephens’ system presents aligned sequences as a graph in which the four basic IUPAC characters (C, T, A, and G) are sized according to the relative probability of finding the corresponding nucleotide at a particular location and stacked to indicate the overall information content encoded by that position. Schneider and Stephen’s successful formula for constructing Sequence Logos was later augmented by Gorodkin et al., who accounted for nucleotide variation across the entire sequence set as well as the possibility of finding gaps in an alignment [7].

**Figure 3:**
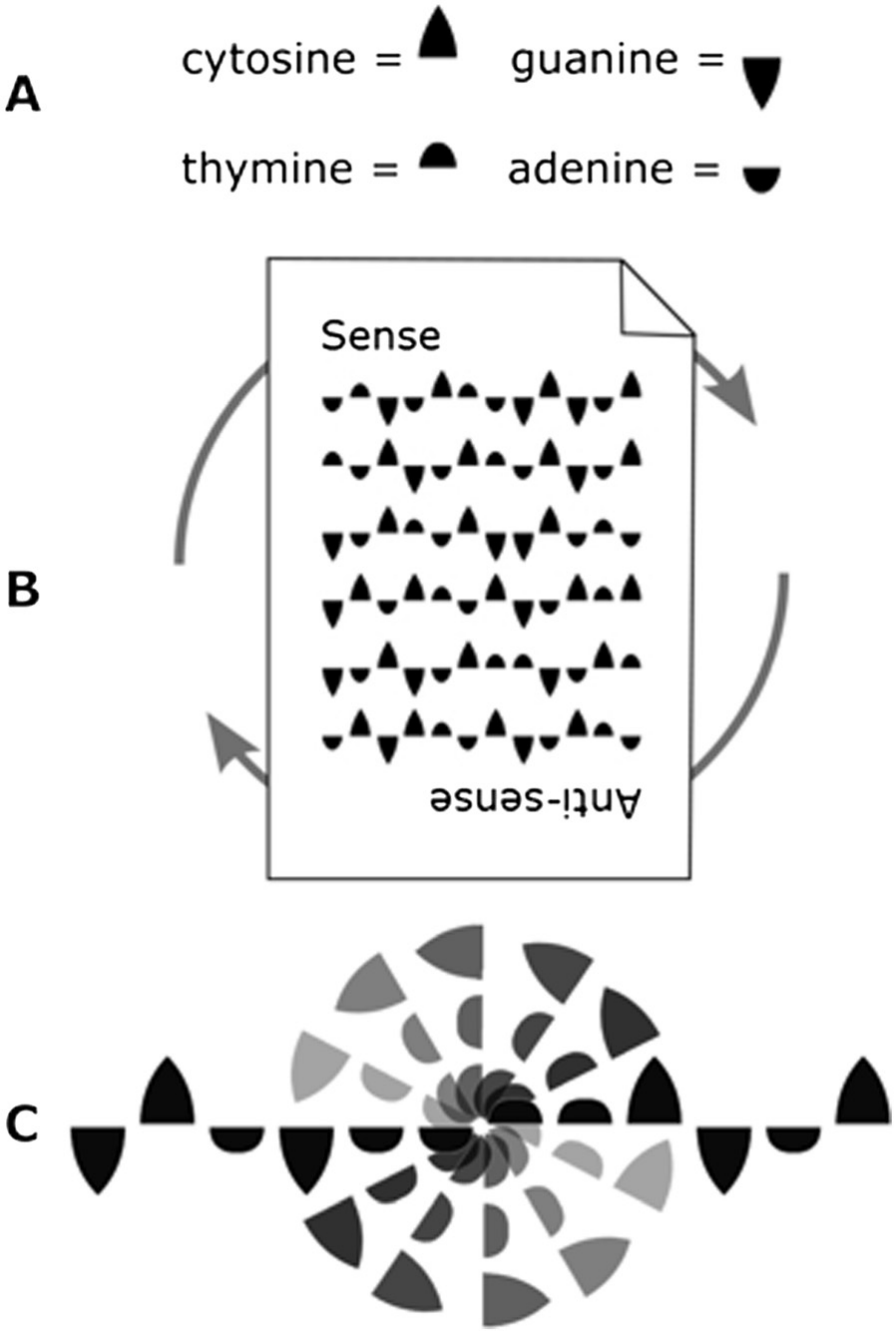
**Ambigraphic nucleic acid notations facilitate the manipulation and analysis of genetic data.** With ambigraphic nucleic acid notations, complementary nucleic acids are represented by symbols that resemble one another when rotated 180 degrees. In the original Ambiscript notation, complementary nucleotides share inverted symbols **(A)**. Consequently, rotating an entire sequence 180 degrees generates its complement **(B)**. Furthermore, palindromes are visually identifiable as stretches of symbols that are unchanged when rotated around a center-point **(C)**.

Our own work with nucleic acid notations is based on the premise that one’s choice of symbol system has immediate cognitive and functional implications for their use. We have argued that using ambigraphic characters to encode nucleic acid sequences facilitates both the manipulation and analysis of genetic data [8]. As defined by Hofstadter in 1985 [9], ambigrams are symbols that convey the same or different meaning when viewed in another orientation. Using a graphically-designed set of nucleic acid symbols [10] we showed that when one represents complementary nucleotides using the same ambigram (Figure 3A), it is possible to derive the complement of an entire sequence by simply rotating the text 180 degrees (Figure 3B). Furthermore, the symmetries inherent in an ambigraphic notation make it easy to recognize palindromic motifs as stretches of characters that can be rotated around a central pivot without changing the appearance of the original sequence (Figure 3C). Our graphically-designed ambigraphic nucleic acid notation, referred to as Ambiscript, also benefits from using symbols that can be overlaid to intuitively form the entire set of ambiguity characters defined by the IUPAC (Figure 2). This feature makes it easy to construct and interpret consensus sequences, overcoming the complexities associated with writing and reading the IUPAC ambiguity code.

Unfortunately, as with IUPAC ambiguity characters, the original Ambiscript notation does not include provisions for encoding the probability of finding a particular nucleotide at a given position in a multiple sequence alignment. Consequently, valuable data is lost when displaying alignments using Ambiscript consensus sequences as well as with IUPAC ambiguity symbols.

Ambiscript Mosaic is a modified version of the Ambiscript notation that permits individual nucleic acid symbols to be represented with colors computed according to the frequencies at which the corresponding nucleotides can be found in each position of a multiple sequence alignment. As with traditional mosaics, where clusters of differently colored glass, ceramic, or stone pieces create a recognizable image when viewed at a distance, the color-coded notation helps users discern biologically-meaningful patterns in nucleic acid sequence alignments.

Ambiscript Mosaic allows users to visually represent and compare sequence polymorphisms in a manner similar to that of the Sequence Logo but requires significantly less space. Furthermore, the color-coded consensus sequences retain all the functional and analytic benefits of the original ambigraphic nucleic acid notation, making it easy to complement sequences and spot biologically relevant palindromes in multiple sequence alignments. The new script also includes a provision for representing gap characters in alignments.

It is important to note that Ambiscript Mosaic is not intended to compete with widely-available computer algorithms, which can quickly and efficiently identify palindromes and other biologically significant patterns in vast tracts of aligned and unaligned sequences. Rather, the graphic display was designed to help convey previously-obtained computational and experimental results in a manner that can be quickly grasped and understood by readers.

Ambiscript Mosaic alignments and consensus sequences can be generated using the online Adobe Flex® application discussed in this article. Unlike the original Ambiscript notation [10], Ambiscript Mosaic displays do not require installation of special computer fonts.

## Implementation

Each of the Ambiscript Mosaic sequence alignments presented in this paper were rendered using the *Ambiscript Mosaic Alignment Tool* (a web-based Adobe Flex® application found at http://www.ambiscript.org), which accepts FASTA-formatted multiple sequence alignments via an internet browser and displays the data as Ambiscript Mosaic consensus sequences. The consensus sequences are rendered by applying the graphic algorithms described below. Both the application and source code are freely available for download from the web site under an open source MIT license. We encourage interested readers to contribute to the code or adapt it for their own use.

## Results and discussion

*Ambiscript Mosaic consensus sequences are constructed by nesting rather than combining nucleotide symbols with figure-ground reversals.* Our original Ambiscript notation provides written and printed symbol sets for flexible use [10]. The DNA Bold font, which uses solid shapes to represent each of the different bases (Figure 2), is functionally suited for encoding information in shades and hues as proposed here. However, a simple design change is needed before the characters can be used to handle color coding.

The original Ambiscript font handled sequence ambiguities with a figure-ground color reversal of the thymine over the cytosine symbol and the adenine over the guanine symbol. This figure-ground color reversal was accomplished by superimposing a white symbol for thymine (a rounded arch) against a black symbol for cytosine (a pointed arch) or a white symbol for adenine (an inverted, rounded arch) over a black symbol for guanine (an inverted, pointed arch) as shown in the Ambiscript DNA Bold column of Figure 2. The Ambiscript Mosaic font, on the other hand, modifies the cytosine and guanine characters so that they have spaces carved out for nesting the smaller thymine and adenine characters. Therefore, in this new system, the possibility of finding a cytosine and thymine at a particular position is denoted by using the new symbol for a cytosine (a pointed arch with a hollowed-out base) and filling its base with the smaller symbol for a thymine (a rounded arch). It is similarly possible to combine inverted, pointed and rounded arches to represent the possibility of finding an adenine and a guanine at the specified location. See the Ambiscript Mosaic column in Figure 2 for examples of how this system is applied to the eleven ambiguity characters.

This new strategy for assembling ambiguity characters has two advantages. First, the open arch further aids the visual distinction between characters. Second, and more importantly, by avoiding the figure-ground reversal used in the original Ambiscript notation, it becomes possible for each of the compounded characters to be colored differently based on the chances of finding the corresponding nucleotide at a given sequence position.

Any graphical representation of complex data requires a degree of practice and familiarity on the part of the reader to effectively perceive and understand the data contained in the graphic. Sequence data rendered via Ambiscript Mosaic is no different in this respect. Nevertheless, we have endeavored throughout our development of the ambigraphic notation to minimize the learning curve for new users by designing symbols that reflect key biological attributes of the nucleotides [10], using compound characters to greatly simplify the construction of ambiguity characters [10], and providing a simple mnemonic to help readers remember the meanings of the four symbols.

A graphic in Figure 2 illustrates how, when the compounded Ambiscript Mosaic characters are read from top to bottom, the first letters of the nucleotide names form the sequence C-TAG. Thus, the meaning of the individual symbols and compounded ambiguity characters is easily recalled by applying the phonetic mnemonic, "sea tag", which in a top to bottom read, links the ambigraphic symbols to the initial letters of the scientific names of the represented nucleotides.

The symbols and principles of Ambiscript Mosaic are such that they can be efficiently described and understood by readers via compact captions and keys, which accompany figures and tables in published manuscripts.

**Figure 4:**
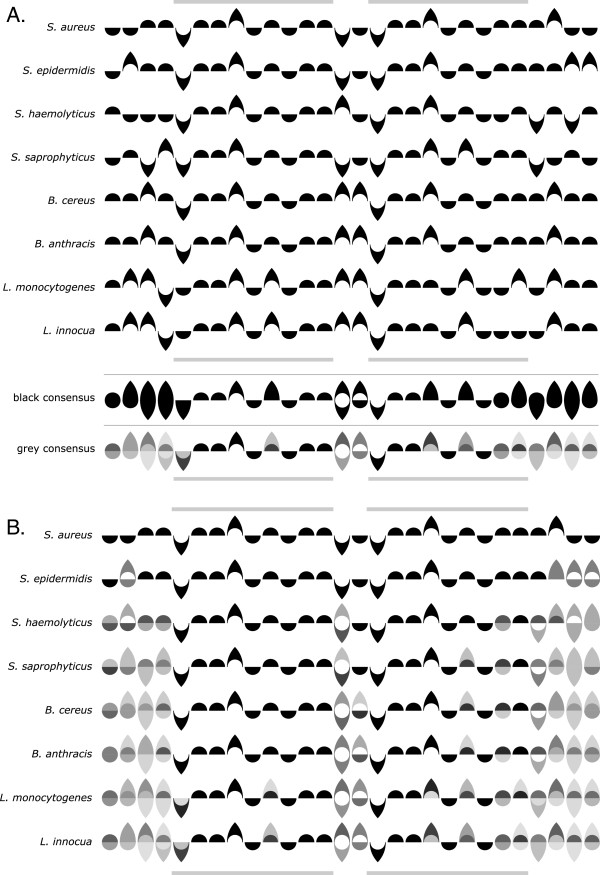
**Ambiscript Mosaic characters can be used to visually encode the degree of genetic variation present in aligned sequence motifs.** Aligned sequences of the common bacterial HssRS DNA binding motif [11] are rendered individually with the black and grey-scaled consensus sequences at the bottom **(A)**. Mosaic consensus symbols are not normally rendered with black-only symbols. The black consensus line shows the limited usefulness of black-only symbols in contrast with the grey shaded symbols in the grey consensus line. Unlike the grey shaded symbols, the black-only symbols are limited to indicating that one or more occurrences of the particular nucleotide can be found in that location, without communicating any sense of the percentage of occurrences. Because Ambiscript Mosaic consensus sequences are more compact than Sequence Logo graphs, they can be displayed using a progressive alignment in which polymorphisms are accumulated as you move down the list, much as numbers are tallied in a financial ledger **(B)**. In both examples, the individual symbols that comprise the compound characters are shaded according to the likelihood of finding them in that particular position of the alignment. The distribution of Ambiscript characters above and below the centerline makes it easy to recognize the uninverted repeat in the aligned motif (bracketed between the grey bars).

*Nested Ambiscript Mosaic symbols can be color-coded to reflect the probabilities of finding different nucleotides at a particular position.* Using solid, nested Ambiscript Mosaic characters to represent ambiguities in consensus sequences allows one to color individual symbols according to the prevalence of the corresponding nucleotide at a given position. Therefore, we have developed an Adobe Flash® application, which uses FASTA-formatted sequence alignments to create Ambiscript Mosaic consensus sequences whose characters are shaded according to their prevalence in the alignment. When using a simple grey-scale display, characters that have a high probability of occurrence are darker, while those that appear infrequently are lightened to fade into the white background. A 100% black symbol represents a 100% chance of finding the indicated nucleotide in that position, while a white symbol (which would not show on a white background) will represent 0% chance of occurrence. Figure 4A shows Ambiscript Mosaic symbols for eight DNA sequences of common bacterial HssRS DNA binding motif [11].

The grey-scaled approach produces two important visual cues. First, the symbols for the most frequently-occurring nucleotides at each position stand out more than the other characters at that location. Second, when the sequence is viewed as a whole, those regions of the genome with less variation are highlighted with dark characters sharply contrasted against the white background. Conversely, those regions with very little sequence homology appear much lighter with lower probabilities of occurrence, and hence lighter shading, evenly distributed among all possible nucleotides. This strategy for shading symbols according to the prevalence of the corresponding nucleotide in multiple sequence alignments provides an intuitive and very effective method for drawing the eye to darker regions of high homology and can be used to efficiently convey genetic disorder in a grey-scaled graphic. Moreover, the white-to-black-coded Mosaic symbols remain compact, allowing multiple consensus sequences to be displayed in a tabular format. Figure 4B takes advantage of this feature by rendering multiple sequences in a progressive format, which shows the accumulation of sequence polymorphisms as each new sequence is added to the list. When the aligned sequences are arranged in order of decreasing homology, the progressive format makes it easier to see the point at which the homologies break down.

*Adding hue and chroma further aids visual discrimination between conserved and variable regions.* It is possible to increase color discrimination in displays that permit more colors than simple mixtures of black and white. This is accomplished by adding hue and chromatic (saturation) perception to luminance (light-to-dark) perception.

The perceptual psychologist, Colin Ware, has observed that greater color sequence discrimination is achieved when hue differences coincide with light-to-dark differences [12]. Therefore, when selecting a color scale for display on computer monitors we looked for one that produced the broadest light-to-dark discrimination that concurs with a pure light primary on one end of the scale.

We also chose to put complementary colors on opposite ends of the scale so that the range would pass through a neutral grey. This allows us to adjust the scale so that the visually distinct neutral grey corresponds to the possibility of randomly finding a nucleotide in a particular location. Depending on the GC-content of the assayed genomes, there is approximately a 25% chance of finding any of the four nucleotides at each position in a randomly-generated sequence. Therefore, any nucleotide that has a 25% chance of occurrence in the displayed sequence alignment is rendered in neutral grey. If all four base symbols are rendered in a neutral grey then it signifies that the alignment lacks apparent consensus at that particular position. Our online application allows users to adjust the scale so that middle grey corresponds with the random distribution of nucleotides in the assayed genome.

Gorodkin’s implementation of Sequence Logo specifically distinguishes nucleotides that occur more or less than random chance by inverting those characters that have a less than random chance of occurrence [7]. This distinction is automatically achieved in Ambiscript Mosaic chromatic scale by recognizing that blue-tinted characters have a greater-than-random occurrence while yellow-tinted characters exhibit a less-than-random occurrence.

When optimizing the color scale for a computer monitor, placing any two of the red, green, or blue LCD light primaries at either end of the scale produces the greatest range of hues between a primary and its complementary hue. However, because none of the three LCD primaries are complementary to one another, they will not produce a neutral grey when mixed in equal amounts. Therefore, one must choose from one of the three primary-derived complementary pairs: red/cyan, green/magenta, or blue/yellow.

As mentioned above, this choice is guided by Ware’s observation [12] that maximum color sequence discrimination is achieved when hue differences coincide with light-to-dark differences. Blue appears to be the darkest of the three full-intensity additive (light) LCD primaries used for digital display because human color vision is least sensitive to this wavelength in the visible electromagnetic spectrum. Furthermore, the particular yellow that is LCD primary blue’s true complement is the lightest hue producible at maximum saturation.

Propitiously, and not accidentally, when Ambiscript Mosaic is rendered with cyan, magenta, yellow, and black (CMYK), process color inks commonly applied in printed publications, yellow is the lightest primary pigment used and its complementary blue, achieved by the mixture of 100% magenta and 100% cyan, will be the darkest of the mixture of any of two of the cyan, magenta or yellow inks.

**Figure 5:**
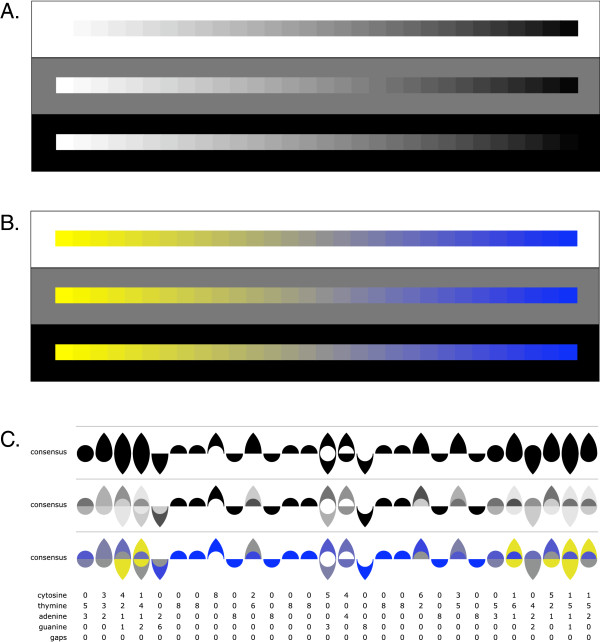
**Using a yellow-to-blue color scale greatly increases the range of visual discrimination.** When white-to-black **(A)** and yellow-to-blue **(B)** color scales are compared against different backgrounds, it is apparent that the latter permits visual discrimination of greater variation, especially at the extremes where the white-to-black scale contrasts with the background. Another advantage of the yellow-to-blue color scale is that the middle grey is distinct from the hues at either extreme and can be used to indicate those bases whose occurrence coincides with random chance. When comparing consensus sequences rendered in the black, grey, and yellow-to-blue color scales **(C)**, sequence variation within the HssRS DNA binding motif [11] is clearest when viewed using the yellow-to-blue scale.

Another advantage of incorporating a hue-chroma scale is that it minimizes the effect of a perceptual phenomena know as contrast crispening. Figure 5A shows a progressive sequence of 30 contiguous grey-scale squares, with computed equal differences ranging from maximum white to maximum black on a digital display system. In this example, it is difficult to discriminate color differences in some regions of the sequence, depending on the color of the background. This phenomenon, in which color differences are perceived as greater when the samples are close in color to the background is called contrast crispening [12]. As shown in Figure 5B, when we add hue and chromatic variations to the sequence color discrimination is significantly increased.

In Figure 5C, we compare consensus sequence data for the HssRS DNA binding motif [11] using the grey scale consensus and the yellow-to-blue scale consensus. In this figure, the absence of a symbol indicates that the corresponding nucleotide is not represented at this particular position of the alignment. With respect to the yellow-to-blue color scale, the lightest and brightest yellow, which tends to blend with the white background, encodes the lowest percentage of occurrences of the signified nucleotide at this position. A neutral middle grey represents 25% occurrences of the signified nucleotide and the darkest and maximum chroma blue, which contrasts most sharply with the white page, represents 100% occurrences of the signified nucleotide at this position.

The Flex application allows for users to select between grey-scales and hue-augmented color scales to encode sequence polymorphisms depending on their personal preferences and the limitations of the particular computer or printed display. However, other color schemes could be developed in future versions of the software, to allow the incorporation of other data sets. For example, Sequence Logo effectively encodes a sequence’s information content in the cumulative heights of the displayed IUPAC characters [13]. It may be possible to render the same information content using a redesigned Ambiscript Mosaic color scale.

**Figure 6:**
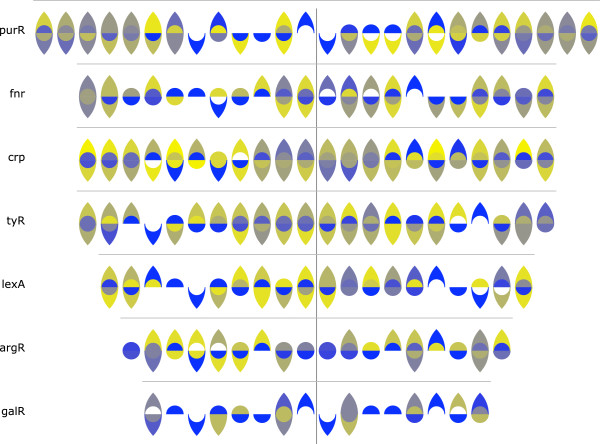
**Ambiscript Mosaic highlights rotational symmetries in sequence alignments.** As illustrated in Figure 3C, symmetries in the Ambiscript notation make it easy to identify palindromic patterns in genetic sequences. These patterns are further highlighted when consensus sequences are shaded and colored according the degree of diversity found among aligned sequence motifs. Consequently, conserved palindromic sequences are easily visualized in many of the *E. coli* binding motifs, previously identified by McGuire et al. [14], as patterns, which remain largely unchanged when rotated around a central pivot (black vertical line).

*Character symmetries make it easy to identify functionally-relevant palindromes in consensus sequences.* The prevalence of dimeric DNA-binding motifs in both prokaryotic and eukaryotic proteomes means that the cognate palindromic nucleotide sequences are frequently conserved within genomes and across species. As noted above, palindromes are easily identified in the original Ambiscript notation as sequence fragments that remain unchanged when rotated around a central pivot (Figure 3C) [10]. Similar rotational symmetries make it particularly easy to spot palindromes in Ambiscript Mosaic consensus sequences, especially when the symbols are shaded and colored-coded based on the degree of genetic variation at each position. To illustrate this feature, we have displayed the consensus sequences for eight previously-described *E. coli* DNA binding motifs [14] using the Ambiscript Mosaic yellow-to-blue color scale (Figure 6). In each of these examples, the dark blue symbols, which represent areas of high genetic conservation in the aligned sequences, are mirrored by the symbols on the other side of the central pivot, indicating the presence of a conserved palindromic pattern.

**Figure 7:**
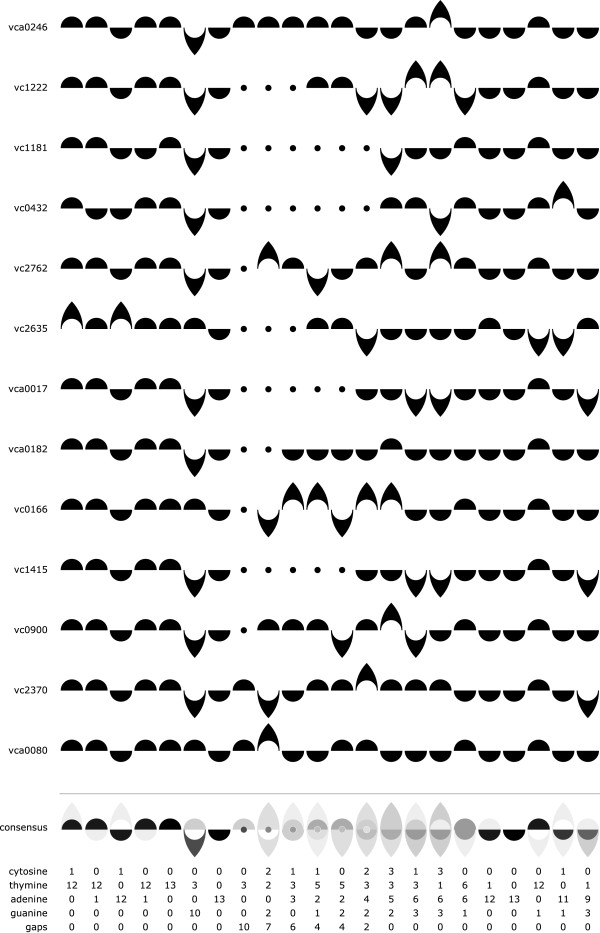
**Ambiscript Mosaic gap characters support the alignment of variably-sized DNA binding motifs.** The consensus sequence generated by aligning members of a *V. cholerae* quorum sensing binding motif 1 [15] reveal both the variable region in the center of the motif and the conserved inverted repeats at either end. Gap characters are rendered as dots, which remain appropriately unchanged when rotated around the center line.

*Adding a gap character supports a broader range of sequence alignments.* Despite the fact that gap characters are frequently found in sequence alignments, very few strategies for rendering consensus sequences explicitly allow for gaps. Following Gorodkin et. al.’s lead of including gap characters in their revised Sequence Logo formulation [7], we have added a gap character to the Ambiscript Mosaic notation that was not present in the original Ambiscript font. Because the complement of a gap is another gap, we have chosen to represent this alignment artifact using a small circle located on the central axis of the compound character. Pivoting the symbols for cytosine, thymine, adenine, or guanine around the central axis produces the symbols for their complements. However, the same rotation leaves the gap character appropriately unaltered. Figure 7 shows how gaps are handled in a *Vibrio cholerae* DNA binding motif reported by Tsou et al. [15].

## Conclusions

The design of our original Ambiscript notation and our subsequent development of Ambiscript Mosaic have been influenced by information design principles proposed by Edward R. Tufte [16]; an understanding of visual semiotics distilled from the writings of Saussure, Pierce, Dunne, and Frank; legibility studies performed by Miles Tinker [17]; and visual perception and cognition research applied by Colin Ware [12] to information design.

The first of Tufte’s principles of graphical excellence is that "graphical excellence consists of complex ideas communicated with clarity, precision, and efficiency" [16]. While the presentations and viewings of genetic data frequently deal with enormous complexity, Tufte’s principles of clarity, precision, and efficiency are poorly served by the IUPAC system of using capital or lower-case Roman letters.

To begin with, the IUPAC notation employs the initial letters of the chemical names for the nucleotides, which if one is aware of the words’ etymologies, vaguely recall the historical source of the chemicals rather than their functional significance within DNA strands. For example, adenine is derived from the Greek word *aden,* which means "gland" and refers to the chemical’s derivation from the pancreas of an ox. Similarly, guanine refers to *guano*, Spanish for "dung", from which the chemical was first isolated. The root for cytosine comes from Greek *kytos* for "a hollow, receptacle, basket". Thymine comes from "thyme", a plant of the mint family, with origins in Greek *thymon*, possibly from *thyein* for "burn as a sacrifice," which would indicate the plant used as incense. While these origins are interesting and may have mnemonic value to chemists and geneticists, they do little to highlight the functional or structural significances of the nucleotides within a DNA strand [18].

**Figure 8:**
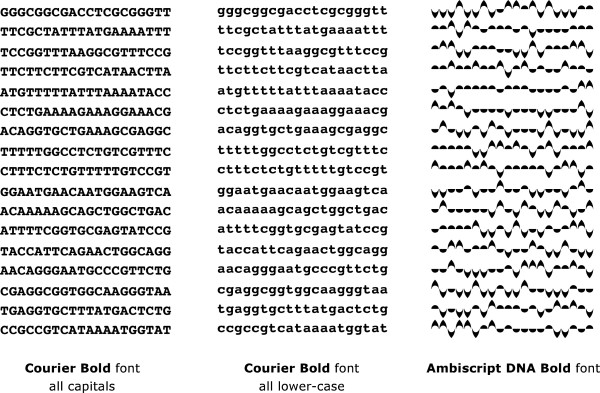
**It is easier to distinguish different Ambiscript symbols than their IUPAC equivalent.** This is because the Ambiscript font relies on ascenders and descenders to distinguish characters, rather than content buried within the x-height of the Roman upper- and lower-case fonts.

Further confounding clarity of the IUPAC notation are the now very arbitrary and obscure references of the Roman letter shapes to the articulatory sources for the sounds they represent. Historically, the Roman characters were derived from pictograms originally voiced in ancient languages, such as the upside-down capital "A" that represented an ox with two horns, and have not been necessarily chosen for their visual clarity, particularly as they have been applied to genetic sequences. Specifically, the mnemonic inadequacy and low legibility of an IUPAC sequence of capital letters (C, T, A, G) or their lower-case variants (c, t, a, g) undermine the potential for rapidly-scanned letter identification and pattern recognition within lengthy DNA sequences. By coincidence and without any intentional design, capital "C" and "G" have similar shapes which could serve to visually reinforce their complementarity. However, within a large sequence of IUPAC letters, the two characters with their aligned baselines and capital top-lines are too similar to be easily distinguished (Figure 8). The Roman capitals "T" and "A", which are visually similar because they contain only straight lines, also establish a weak visual correlation between the two complementary nucleotides. However, when rendered as capitals and especially when set in small point sizes, the four IUPAC letters lose their visual distinctiveness because they are all the same height and evenly aligned at their top and bottom ends, visually obscuring relevant DNA patterns.

In contrast, the use of lower-case "c", " t", "a", " g" provide more readily distinguished patterns because of the degree to which the top of the "t" and the descender of the "g" extend above and below the horizontal space created by the "x" height portions of the four letters (Figure 8). Unfortunately, other visual cues, which could facilitate the identification of meaningful genetic patterns, are missing. For example, the "c" for cytosine does not have an ascender stroke to match the descender stroke on the "g" representing guanine. If the "t" did not have an ascender, it would better serve as a complement for the "a" representing adenine.

Comparing three different settings of the same genetic sequence, one with upper-case Roman characters, another with lower-case Roman characters and a third with Ambiscript Mosaic, all within the same-sized space, should demonstrate the much-improved clarity achieved with the Ambiscript Mosaic notation (Figure 8). Here, sequences of stronger and weaker nucleotide bonds, as well as sequences of heavier verses lighter nucleotides, and their resulting patterns are clearly evident, making it easier to locate palindromes. In contrast to type faces such as Garamond, which has an "x" height of 39% of the font’s type size, and Helvetica, which has an "x" height that is 52% of the font’s type size, we deliberately chose to remove the "x" height from Ambiscript. This eliminates the risk of burying any important character features within a high-density "x" height region. Instead, distinctive visual features appear above the horizontal baseline as ascenders and below the baseline as descenders. Fortunately, this high degree of legibility is much easier to accomplish for the four DNA symbols, than for the 26 lowercase Roman letters.

Recent studies in perception and cognition enable more accurate discussion of the differences between processing symbolic and non-symbolic signs. Symbols are socially approved signs, as opposed to sensory representations, such as size differences and color variations that are non-symbolic signs. Researchers, such as Colin Ware, refer to these non-symbolic signs as "perceptual representations" [12]. In developing our original Ambiscript symbols, we employed perceptual representations as much as we could to avoid the arbitrary signifiers (such as the historically remote use of pictographs to represent articulatory sounds and the entomologically obscure names for nucleotides) that are often required to overcome limits in human perception. Our use of color-coding applies additional perceptual representation (non-symbolic signs) to our solution.

Another of Tufte’s principles is that "graphical excellence is nearly always multivariate" [16]. To meet this criterion, displayed data should enable the simultaneous observation and the analysis of more than one data variable. We believe that we have made significant progress towards this standard by reconfiguring the Ambiscript characters to allow color-coded representations of genetic variation among multiple sequence alignments.

The figure-ground reversals used in our original Ambiscript notation enabled us to give readers "the greatest number of ideas in the shortest time with the least ink in the smallest space" [16]. Consequently, a single 8 point, black-on-white, ambiguity symbol can convey the presence of two, three or four different nucleotides in this particular position for as many DNA sequences as are being compared. However, our original design for Ambiscript ambiguity characters did not permit us to represent the frequency of each of these different nucleotide bases in any single location, as would be highly useful for a number of DNA analyses.

Fortunately, by using nested, rather than silhouetted symbols to construct consensus sequences, we can visually represent distributions of nucleotide polymorphisms using colors and shadings so that viewers can more easily perceive these patterns. The loss here is that human perception does not permit legible Ambiguity Mosaic symbols as small as 8 point type. At such small sizes color-coding becomes very unreliable because of what Ware explains as the occurrence of small-field color tritanopia, the inability to distinguish colors that are different in the yellow-blue direction [12]. Not only this, color print reproduction and resolution at such small type sizes is questionable. However, the tradeoff benefit is the significant increase of information gained by the color-coded symbols.

**Figure 9:**
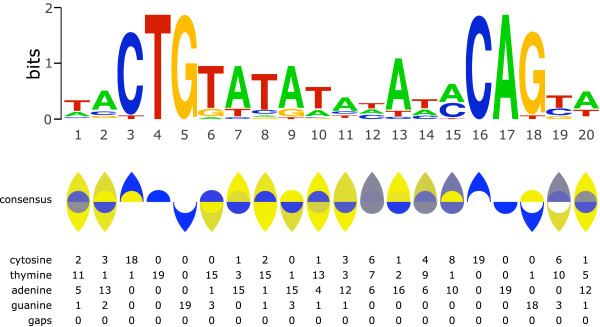
**Ambiscript Mosaic allows biologists to render sequence data using less space and with greater clarity than a Sequence Logo graph.** Despite being half the height of a comparable Sequence Logo graph, the Ambiscript Mosaic characters can be used to similarly highlight conserved nucleotides in the *E. coli* lexA binding motif reported by McGuire et al. [14]. This is accomplished without degrading the legibility of infrequently-represented nucleotides. More importantly, the physical symmetries of the ambigraphic notation make it easy to spot the palindromic regions at either end of conserved binding motif.

Ambiscript Mosaic and Sequence Logo represent alternate realizations of Tufte’s principle that "graphical excellence is that which gives to the viewer the greatest number of ideas in the shortest time with the least ink in the smallest space" [16]. We believe that Ambiscript Mosaic has three advantages over the widely-used Sequence Logo display. All three of these aspects are illustrated in the Figure 9 side-by-side comparison of the two systems. However, it should be noted that that this comparison specifically addresses the graphical features of the two systems and not the information content computations, which are encoded by the Sequence Logo display [6,7,13,19] and may potentially be incorporated into future versions of the Ambiscript Mosaic software.

First, sequences rendered in Ambiscript Mosaic require only a fraction of the space generally used for Sequence Logo displays. Even at half the size presented here, Ambiscript Mosaic is still very readable, while at that further reduction, Sequence Logo is that much less legible. This becomes an important feature when the number or size of the reported consensus sequences increases. It also makes it possible to compare multiple consensus sequences in tables or lists similar to the examples found in Figure 6. While Sequence Logos can and have been rendered in more compact spaces [14], this cannot be accomplished without loosing resolution on less than highly conserved regions of the sequence. Ambiscript Mosaic does not entail this sacrifice. Nevertheless, Ambiscript Mosaic’s compactness comes at a cost. It is harder to visually extrapolate precise values from a color scale than from the height-modulated characters used in Sequence Logo.

The second advantage that Ambiscript Mosaic offers over Sequence Logo is that the notation provides a more visually and conceptually coherent and attractive solution. It avoids the typographically awkward distortions that occur with the excessive vertical stretching and shrinking of letter-forms used for Sequence Logo, which not only produces unattractive typography, but also renders illegible variations.

The third and most important advantage Ambiscript Mosaic offers users is that the inherent symmetries found in the ambigraphic notation allow the reader to rapidly spot palindromic patterns that are often obscured in the IUPAC-derived presentation. This new functionality is available via a simple application, which can be downloaded or accessed online via Adobe Flash®-capable internet browsers at http://www.ambiscript.org.

## Availability and requirements

• **Project name:** Ambiscript Mosaic

• **Project home page:**http://www.ambiscript.org

• **Operating system(s):** Platform independent

• **Programming language:** Adobe Flex®

• **Other requirements:** An Adobe Flash®-enabled internet browser

• **License:** MIT License

## Competing interests

The authors declare that they have no competing interests.

## Authors’ contributions

DAR defined the visual and functional requirements for the nucleic acid notation. AJR was responsible for the physical design and shape of the symbols. Both authors made significant contributions to the Adobe Flex® code and have read and approved the final manuscript.
